# Exploratory Evaluation of a Medical Student‐Led Health Literacy Program Among High School Students

**DOI:** 10.1111/josh.70220

**Published:** 2026-08-20

**Authors:** Noah Lee Carey, Isabella Johnson, Allison Ruff, Jonathan F. Finks

**Affiliations:** ^1^ Department of Surgery, Medical School University of Michigan Ann Arbor Michigan USA; ^2^ Department of Internal Medicine, Medical School University of Michigan Ann Arbor Michigan USA

**Keywords:** adolescent health, community engagement, health equity, health literacy, medical education, social determinants of health

## Abstract

**Background:**

Low health literacy is a barrier to positive health behaviors and outcomes among adolescents, especially in marginalized communities. Sustained, youth‐centered interventions for high‐risk populations are limited.

**Contributions to Practice:**

We implemented and conducted an exploratory evaluation of a year‐long, medical student‐led program for ninth‐ and tenth‐grade students at two Detroit public high schools. The program demonstrated the feasibility of interactive school‐based health literacy programming and identified practical considerations for future implementation. Using anonymous pre and postprogram surveys, we examined group‐level response patterns in self‐reported confidence on health‐related knowledge and abilities (*n* = 71 baseline; *n* = 52 follow‐up). Survey items reflected domains addressed during program sessions, including healthcare navigation, understanding medical terminology, identifying reliable health information, self‐advocacy in healthcare settings, and emergency response skills. Postsurvey responses showed more favorable group‐level response patterns across all domains.

**Implications for School Health Policy, Practice, and Equity:**

Near‐peer, school‐based health literacy interventions may represent a practical strategy to support adolescent engagement with healthcare concepts in under‐resourced communities. Partnerships between medical schools and secondary schools may provide scalable opportunities for community engagement and health education.

**Conclusions:**

This exploratory evaluation supports the potential value of medical student‐led health literacy programming as a community‐centered model for adolescent health education.

## 1 Introduction

Health literacy is a key driver of adolescent health and wellbeing [1]. Distinct from general literacy, which refers to the fundamental ability to read, write, speak, comprehend, and use basic math, health literacy emphasizes the application of such skills in health‐related contexts [2]. It encompasses the ability to locate reliable health information and services, understand medical instructions and related documentation, critically evaluate the quality and relevance of health information, and make informed health decisions [3]. Many adolescents may face barriers to developing adequate health literacy because these competencies rely on foundational literacy skills [4]. In a study of ninth‐grade students in an urban school district, nearly half were reading two or more grades below expected levels [5]. Among these students, lower health literacy scores were associated with lower self‐ratings of general health, unhealthy dietary patterns, heavier weight, and increased engagement in risk behaviors. The student population in this study reflected diverse racial, ethnic, and socioeconomic backgrounds, underscoring how structural inequities contribute to intersecting disparities in health literacy and other social determinants of health. Similarly, another study among Appalachian middle school students demonstrated that 47% had limited health literacy, which was associated with lower consumption of fruits, vegetables, and water, higher intake of sugar‐sweetened beverages and junk food, increased screen time, higher BMI percentiles, and lower quality of life [6]. These findings align with broader evidence that health literacy follows a social gradient, with youth from lower socioeconomic backgrounds and marginalized racial and ethnic groups disproportionately affected by limited health literacy [7, 8, 9].

Despite this evidence, few interventions have been created with the aim of directly improving health literacy among disadvantaged youth [10], underscoring the need for targeted efforts to address these inequities and reduce associated health disparities. Schools represent a particularly appropriate setting for such interventions, as they provide near‐universal access to adolescents across socioeconomic contexts and offer a structured environment for delivering sustained educational programming during a critical developmental period for health behavior formation and healthcare navigation skills [11]. In this context, medical schools represent a potentially valuable partner in efforts to improve adolescent health literacy. Across the United States, medical schools provide access to facilities and equipment that can be used for educational demonstrations, student organizations that engage in outreach to help train individuals in life‐saving procedures (e.g., naloxone administration), and labor forces made up of students from a variety of backgrounds who can lead small‐group teaching sessions [12, 13, 14, 15, 16, 17]. This arrangement may be mutually beneficial for high school students and medical students alike, as high school students gain confidence in navigating a complex healthcare system and medical students practice teaching and building rapport with members of their community, fundamental skills of a physician.

To address this need, we developed a year‐long, medical student‐led health literacy initiative for students attending public high schools in Detroit. The program included interactive sessions focused on practical health navigation skills and hands‐on training in emergency response interventions, such as naloxone administration. In addition to providing exposure to health‐related concepts, the program sought to support students' confidence and perceived preparedness in navigating healthcare‐related situations.

As part of an exploratory program evaluation, anonymous surveys were administered before and after program participation to assess group‐level response patterns related to self‐reported confidence and perceived ability across healthcare‐related domains. Because surveys were anonymous and respondents could not be linked longitudinally, the evaluation was intended to provide pragmatic, implementation‐focused insights rather than establish individual‐level change or intervention efficacy. The primary objective of this paper is to describe the implementation of this school‐based, medical student‐led health literacy initiative and explore patterns in pre‐ and postprogram survey responses related to perceived confidence in healthcare navigation, emergency response preparedness, and health‐related skills among participating students.

## 2 Presentation of the Practice

### 2.1 Program Context and Setting

The health literacy program was implemented for students from Cass Technical High School and the School at Marygrove through an existing outreach partnership with the University of Michigan Medical School's *Doctors of Tomorrow* program, which aims to expose students from historically underrepresented backgrounds to careers in medicine and healthcare. The health literacy program was introduced as a pilot initiative within these partner schools that had known student interest in health‐related enrichment opportunities and the logistical capacity to support recurring sessions throughout the academic year. All ninth‐ and tenth‐grade students enrolled in the 2023–2024 academic year who were involved in *Doctors of Tomorrow* were eligible to participate.

Individual‐level demographic data were not collected from program participants; therefore, institutional‐level data are provided to describe the broader school contexts rather than the analytic sample. Aggregate demographic characteristics were obtained from publicly available sources. At Cass Technical High School, approximately 74% of students identified as Black or African American, 13% as Hispanic or Latino, 10% as Asian, and 2% as White [18]. 60.6% of the students at Cass Technical High School were identified as economically disadvantaged. At the School at Marygrove, approximately 98% of students identified as Black or African American, and 67.4% of the students were identified as economically disadvantaged.

### 2.2 Program Description

The health literacy program consisted of six approximately 1‐h instructional sessions delivered over an 8‐month period. Educational content was derived from a combination of adapted, preexisting curriculum materials and original content developed by the program team. Specifically, selected modules were adapted from a publicly available facilitator guide developed by Nemours Children's Health, which was chosen for its evidence‐informed, age‐appropriate health education content designed to improve adolescent health literacy [19, 20]. Additional lessons were designed by medical student facilitators to align with program objectives and the local community context.

All instructional materials were intentionally delivered at an age‐appropriate and accessible level, with emphasis on plain language, visual aids, and interactive activities to accommodate varying literacy levels. Sessions incorporated interactive lectures, small‐group activities, and case‐based discussions focused on health system navigation, emergency response, and preventive care, with the goal of improving both health knowledge and self‐efficacy in accessing and using health information. Specific session topics were healthcare system navigation and use of care settings (primary care, urgent care, and emergency care); health management, nutrition, and physical activity; identifying reliable health information and self‐advocacy in healthcare encounters; common medical terminology; health insurance; and opioid medication safety, overdose recognition, and naloxone administration.

The program was delivered in person by medical student facilitators. Facilitators were selected from a pool of first‐year medical student applicants through a written application and interview process led by *Doctors of Tomorrow* leadership. Prior experience in teaching, mentorship, and educational content development was highly valued. Preparation for program delivery included review of structured lesson plans and session‐specific materials, along with orientation to the participating student population and school context. Participants were encouraged to attend all six sessions; however, attendance varied due to scheduling and logistical constraints. As a result, sessions were attended by overlapping but nonidentical groups of students, and not all participants attended every session.

Implementation was influenced by common logistical constraints in school‐based programming, including variable attendance and academic‐year scheduling limitations, which affected session continuity and participation. One notable strength of the program was collaboration with an existing medical student organization providing naloxone education, which contributed content expertise and facilitated hands‐on naloxone administration training. However, coordination across multiple facilitator groups also introduced scheduling and organizational challenges. The program also pursued collaboration with another medical student‐led organization to incorporate cardiopulmonary resuscitation (CPR) training. Although the necessary expertise and volunteer support were available, the organization's multisession training model could not be accommodated within the program's schedule. Future iterations with greater scheduling flexibility may be able to leverage this established partnership to expand the curriculum with CPR instruction.

Facilitators observed that students appeared particularly receptive to interactive and applied learning activities. Future programming may benefit from incorporating similar approaches into less applied content areas, such as medical terminology. Improved coordination across student groups and continued use of external partnerships may further strengthen experiential learning opportunities. Overall, these findings suggest that successful replication may benefit from flexible scheduling, established school partnerships, and adaptable, partnership‐supported session design.

### 2.3 Exploratory Evaluation Approach

A structured survey was administered immediately before (presurvey) and after (postsurvey) program participation. The survey consisted of attitudinal measures assessed with Likert‐scale items (strongly disagree, disagree, undecided, agree, and strongly agree) and was newly designed as an evaluation tool to inform iterative curriculum development by identifying higher or lower student‐reported confidence and understanding. As a result of this intention, there was no formal psychometric validation. Survey items assessed self‐reported confidence and perceived ability across selected health‐related domains: healthcare navigation, understanding of medical terminology, identifying reliable health information, self‐advocacy in healthcare settings, and emergency response skills. The same items were used at both time points, and the complete survey is provided in Table S1. Item ordering generally followed the sequence of curricular topics delivered throughout the academic year. Because the postsurvey was administered after program completion, responses may have been influenced by recency bias, particularly for topics delivered later in the curriculum. Surveys were paper‐based. Participation in the surveys was voluntary and anonymous. No personally identifying information was collected, and de‐identified tracking codes were not assigned. Completed surveys were collected by program staff, manually transferred into a database, and checked for completeness prior to analysis.

Given the exploratory design, analyses are presented as group‐level descriptive comparisons. Likert‐scale responses were collapsed into agree/strongly agree versus all other response categories for analysis. Pre‐ and postprogram differences at the group level were analyzed using contingency tables. Due to small cell counts on several survey items, Fisher's exact test was used in place of chi‐squared tests to assess changes in categorical responses. Results are presented as distributions across response categories. Analyses were conducted using the statistical software R (version 2025.05.1 + 513), with significance set at *p* < 0.05.

### 2.4 Survey Response Patterns

A total of 71 students completed the presurvey, and 52 completed the postsurvey. Across survey items, postsurvey responses demonstrated favorable group‐level response patterns in all assessed domains (Table 1).

**TABLE 1:** Comparison of pre‐ and postintervention categorical survey responses using Fisher's Exact Test.

Survey item	Pre: agree/strongly agree *n* (%)	Post: agree/strongly agree *n* (%)	*p*
1. I know what services a Primary Care Provider can help me with.	46/71 (64.79%)	49/52 (94.23%)	< 0.001
2. I am confident that I can identify what makes up a healthy diet.	64/71 (90.14%)	49/52 (94.23%)	0.516
3. If someone is overdosing on an opioid, I am confident that I know how to help them.	15/69 (21.74%)	38/52 (73.08%)	< 0.001
4. I am confident in recognizing the symptoms of an opioid overdose.	21/70 (30.00%)	42/51 (82.35%)	< 0.001
5. I can explain what a PPO is.	7/71 (9.86%)	16/52 (30.77%)	0.005
6. I can effectively explain what a deductible is.	11/70 (15.71%)	24/52 (46.15%)	< 0.001
7. I know what adeno‐ means medically.	6/70 (8.57%)	11/52 (21.15%)	0.064
8. I know what‐megaly means medically.	8/70 (11.43%)	11/52 (21.15%)	0.206
9. I can confidently find reputable sources of medical and health information.	50/71 (70.42%)	50/52 (96.15%)	< 0.001
10. I can advocate for myself and my medical needs when meeting with my care team.	55/71 (77.46%)	49/52 (94.23%)	0.012

*Note:* Denominators vary across items due to exclusion of responses with multiple selections or no response, which were considered invalid for item‐level analyses. Nominal *p*‐values are presented without adjustment for multiple comparisons.

The largest differences in agree/strongly agree responses were observed for items related to opioid overdose response preparedness and healthcare system knowledge. Confidence in helping someone experiencing an opioid overdose increased from 21.74% preprogram to 73.08% postprogram (*p* < 0.001), while confidence in recognizing opioid overdose symptoms increased from 30.00% to 82.35% (*p* < 0.001). The proportion of students reporting confidence in identifying reputable health information sources increased from 70.42% to 96.15% (*p* < 0.001), and confidence in understanding services provided by a primary care provider increased from 64.79% to 94.23% (*p* < 0.001).

More moderate increases were observed for items assessing familiarity with healthcare terminology and insurance concepts. Agreement with the statement “I can explain what a PPO is” increased from 9.86% to 30.77% (*p* = 0.005), while confidence in explaining what a deductible is increased from 15.71% to 46.15% (*p* < 0.001).

The item assessing self‐advocacy in healthcare settings showed a smaller but still favorable shift in response distribution, with agree/strongly agree responses increasing from 77.46% to 94.23% (*p* = 0.012). The item assessing confidence in identifying components of a healthy diet showed minimal change between surveys (90.14% vs. 94.23%; *p* = 0.516).

## 3 Discussion

This paper describes the implementation and exploratory evaluation of a medical student‐led health literacy program for public high school students in Detroit. Program implementation highlighted the practicality of interactive, partnership‐supported health literacy education within school settings while identifying important considerations for replication including flexible scheduling, sustained school engagement, and coordination across collaborating organizations. Follow‐up survey responses from participants demonstrated more favorable group‐level response patterns across several domains related to healthcare navigation, emergency response preparedness, and healthcare self‐advocacy.

### 3.1 Implications for School Health Research

Most health literacy research has focused on adult populations, with comparatively little attention paid to adolescents [1]. The COVID‐19 pandemic highlighted the need for adolescents to effectively interpret changing health information and navigate healthcare systems [21]. Our exploratory evaluation of this program suggests that high school‐based, medical student‐led approaches may improve adolescents' confidence in self‐advocacy and healthcare navigation skills that could support long‐term engagement with healthcare systems into adulthood.

Emerging literature suggests that near‐peer educational models operate through mechanisms extending beyond information delivery alone. A recent realist‐informed systematic review of school‐based peer education interventions identified several factors associated with successful implementation, including perceptions of facilitator credibility and relatability (i.e., “peerness”), a balance between autonomy and support, alignment with school culture and values, and informal, personalized approaches to content delivery [22]. This program intentionally incorporated several of these elements through interactive small‐group sessions, applied learning activities, and instruction delivered by medical student facilitators positioned closer in age and social experience to participating adolescents than traditional authority figures. These characteristics may be particularly valuable in adolescent health literacy interventions, where comfort discussing sensitive topics and willingness to ask questions can influence participation and learning outcomes.

The facilitator role in this intervention also aligns with research examining how students in health professions understand and engage with health literacy. A recent cross‐country study among nursing students found substantial variability in familiarity with and conceptualizations of health literacy, with some students emphasizing basic health knowledge and others focusing more on healthcare navigation, communication, prevention, and practical decision‐making [23]. These findings suggest that trainees in health professions may enter health education settings with differing levels of preparation and perspectives regarding patient communication and public engagement. In this program, medical students served not only as content facilitators but also as translators of complex health concepts into accessible, developmentally appropriate, and community‐relevant language. This model may therefore provide reciprocal educational value by supporting adolescent engagement while simultaneously allowing trainees to more actively engage with health literacy as a practical and professionally relevant component of future clinical practice.

Future research could incorporate unique identifiers to enable individual‐level analyses, expand sample sizes and schools to evaluate scalability, assess whether improvements in confidence translate into healthier behaviors over time, and examine how near‐peer teaching influences medical student outcomes, including communication skills, cultural competence, and commitment to community engagement. Such research would provide evidence to guide the design, implementation, and evaluation of school‐based health literacy programs and inform best practices for similar educational interventions.

### 3.2 Implications for School Health Policy, Practice, and Equity

Previous studies have evaluated the prevalence and consequences of low health literacy among adolescents, demonstrating associations with poorer health outcomes, less healthy behaviors, and reduced healthcare engagement [1, 24]. Our intervention builds on this work by describing the implementation of a pragmatic, school‐based educational program and examining group‐level patterns in self‐reported confidence and perceived ability across healthcare‐related domains.

From a school health practice perspective, the findings suggest that interactive health education delivered in partnership with medical student facilitators is feasible to implement within high school settings. With respect to school health equity, these findings should be interpreted as descriptive evidence of how students in under‐resourced community settings responded to structured health education. The observed engagement with emergency response content (e.g., overdose recognition and naloxone awareness) highlights the potential value of including applied, safety‐oriented health topics within school‐based curricula. Near‐peer, medical student‐led programming may also offer benefits beyond student learning, supporting structured opportunities for medical trainees to develop communication skills and experience in community‐engaged education.

### 3.3 Limitations

The study should be interpreted in the context of multiple limitations. First, pre‐ and postsurvey responses were not linked using de‐identified tracking codes, which precluded paired individual‐level analyses and limited interpretation to group‐level comparisons. Importantly, baseline and follow‐up samples were not identical, as attendance varied across sessions and the postsurvey reflects an overlapping but potentially nonequivalent subset of students. This introduces the possibility of selection effects, including the likelihood that students who remained engaged in the program or who felt more confident were more likely to complete the postsurvey, potentially biasing observed response patterns. In addition, relatively small cell counts for several survey items necessitated the use of Fisher's exact test rather than chi‐squared testing. Although Fisher's test is appropriate for small samples, it may reduce statistical power and limit the ability to detect more subtle differences. Given the exploratory nature of the evaluation and the number of item‐level comparisons performed, statistical results should be interpreted as descriptive and hypothesis‐generating rather than confirmatory, and no adjustment for multiple comparisons was applied. Finally, Likert‐scale data were analyzed as categorical rather than ordinal or continuous, which limits the ability to assess gradations in response change and may obscure more nuanced shifts in student perceptions.

Despite these limitations, our findings have several implications for school‐based health promotion and the implementation of near‐peer educational programming. They suggest that partnerships between academic health centers and secondary schools can support the delivery of structured, interactive health education using medical student facilitators within resource‐constrained school settings. More broadly, this model highlights the potential value of near‐peer approaches in expanding access to applied health education.

In summary, this exploratory evaluation describes a feasible strategy for delivering health literacy programming through medical‐student facilitators and provides descriptive evidence of positive group‐level shifts in self‐reported confidence across selected health‐related domains among high school students. Future iterations may benefit from incorporating additional opportunities for applied skill‐building, leveraging broader institutional resources within the academic setting, and expanding the program's reach to additional high schools. A more robust evaluation method would allow for a more rigorous assessment of program effectiveness, including the ability to evaluate sustained changes over time and strengthen conclusions regarding impact. Our findings support the value of sustained academic–secondary school partnerships in facilitating scalable, medical student‐led approaches to school‐based health literacy education.

## 4 Conclusions

This exploratory evaluation of a medical student‐led health literacy program demonstrated favorable postsurvey group‐level response patterns across multiple domains related to healthcare navigation, emergency response preparedness, and healthcare self‐advocacy among participating adolescents. These findings provide preliminary, practice‐oriented insights into the feasibility and potential promise of interactive, youth‐centered health literacy programming within school settings, particularly in historically marginalized communities facing barriers to health education and care access. The program also highlighted practical considerations relevant to implementation and replication, including the value of flexible scheduling, sustained partnerships, and applied learning activities.

## Funding

This work was supported by the President Ono's Inauguration Poster Session Award at the University of Michigan.

## Ethics Statement

This study was reviewed and exempted by the University of Michigan Institutional Review Board as an educational program evaluation (HUM00240655). All participants were informed of the voluntary and anonymous nature of the survey.

## Conflicts of Interest

The authors declare no conflicts of interest.

## Supporting information


**Table S1:** Sample pre‐ and postintervention survey.

## Data Availability

The data that support the findings of this study are available on request from the corresponding author. The data are not publicly available due to privacy or ethical restrictions.

## References

[1] S. A. Fleary , P. Joseph , and J. E. Pappagianopoulos , “Adolescent Health Literacy and Health Behaviors: A Systematic Review,” Journal of Adolescence 62 (2018): 116–127, 10.1016/j.adolescence.2017.11.010. 29179126

[2] Ad Hoc Committee on Health Literacy for the Council on Scientific Affairs AMA , “Health LiteracyReport of the Council on Scientific Affairs,” Journal of the American Medical Association 281, no. 6 (1999): 552–557, 10.1001/jama.281.6.552. 10022112

[3] odphp.health.gov , “Health Literacy in Healthy People 2030—Healthy People 2030,” accessed December 31, 2025, https://odphp.health.gov/healthypeople/priority‐areas/health‐literacy‐healthy‐people‐2030.

[4] J. Warsh , R. Chari , A. Badaczewski , J. Hossain , and I. Sharif , “Can the Newest Vital Sign be Used to Assess Health Literacy in Children and Adolescents?,” Clinical Pediatrics 53, no. 2 (2014): 141–144, 10.1177/0009922813504025. 24065737

[5] A. Park , T. L. Eckert , M. J. Zaso , et al., “Associations Between Health Literacy and Health Behaviors Among Urban High Schoolers,” Journal of School Health 87, no. 12 (2017): 885–893, 10.1111/josh.12567. 29096408 PMC5669371

[6] A. L. Reid , K. J. Porter , W. You , et al., “Low Health Literacy Is Associated With Energy‐Balance‐Related Behaviors, Quality of Life, and BMI Among Rural Appalachian Middle School Students: A Cross‐Sectional Study,” Journal of School Health 91, no. 8 (2021): 608–616, 10.1111/josh.13051. 34096052 PMC9660538

[7] D. Nutbeam and J. E. Lloyd , “Understanding and Responding to Health Literacy as a Social Determinant of Health,” Annual Review of Public Health 42 (2021): 159–173, 10.1146/annurev-publhealth-090419-102529. 33035427

[8] M. Duplaga and M. Grysztar , “Socio‐Economic Determinants of Health Literacy in High School Students: A Cross‐Sectional Study,” International Journal of Environmental Research and Public Health 18, no. 22 (2021): 12231, 10.3390/ijerph182212231. 34831987 PMC8624924

[9] K. A. Kaphingst , M. Goodman , O. Pyke , J. Stafford , and C. Lachance , “Relationship Between Self‐Reported Racial Composition of High School and Health Literacy Among Community Health Center Patients,” Health Education & Behavior: Official Publication of the Society for Public Health Education 39, no. 1 (2012): 35–44, 10.1177/1090198111406538. PMC3170677 21636703

[10] C. Smith , H. R. Goss , J. Issartel , and S. Belton , “Health Literacy in Schools? A Systematic Review of Health‐Related Interventions Aimed at Disadvantaged Adolescents,” Children 8, no. 3 (2021): 176, 10.3390/children8030176. 33668861 PMC7996245

[11] M. D. Wong , K. H. Quartz , M. Saunders , et al., “Turning Vicious Cycles Into Virtuous Ones: The Potential for Schools to Improve the Life Course,” Pediatrics 149 (2022): e2021053509M, 10.1542/peds.2021-053509M. PMC9113000 35503311

[12] S. A. Saberi , S. Moore , S. Li , et al., “Systemized Approach to Equipping Medical Students With Naloxone: A Student‐Driven Initiative to Combat the Opioid Crisis,” BMC Medical Education 24, no. 1 (2024): 241, 10.1186/s12909-024-05221-8. 38448949 PMC10916177

[13] E. N. Waskel , S. C. Antonio , G. Irio , J. L. Campbell , and J. Kramer , “The Impact of Medical School Education on the Opioid Overdose Crisis With Concurrent Training in Naloxone Administration and MAT,” Journal of Addictive Diseases 38, no. 3 (2020): 380–383, 10.1080/10550887.2020.1762030. 32449488

[14] L. E. Nagle , T. E. H. Moses , A. Chitale , et al., “Building a Strong Foundation From the Ground Up: The Impact of a Medical Student Substance Use Disorder Organization on Curriculum and Community,” Journal of Addictive Diseases 41, no. 2 (2023): 156–166, 10.1080/10550887.2022.2068907. 35470767 PMC9745562

[15] K. Karpa , K. Vakharia , C. A. Caruso , C. Vechery , L. Sipple , and A. Wang , “Medical Student Service Learning Program Teaches Secondary Students About Career Opportunities in Health and Medical Fields,” Advances in Physiology Education 39, no. 4 (2015): 315–319, 10.1152/advan.00124.2015. 26628654

[16] H. J. Doyle , “UCSF Partnership to Enrich Science Teaching for Sixth Graders in San Francisco's Schools,” Academic Medicine: Journal of the Association of American Medical Colleges 74, no. 4 (1999): 329–331, 10.1097/00001888-199904000-00015. 10219201

[17] T. W. Vijn , C. R. M. G. Fluit , J. A. M. Kremer , T. Beune , M. J. Faber , and H. Wollersheim , “Involving Medical Students in Providing Patient Education for Real Patients: A Scoping Review,” Journal of General Internal Medicine 32, no. 9 (2017): 1031–1043, 10.1007/s11606-017-4065-3. 28600753 PMC5570739

[18] Michigan's Center for Educational Performance and Information , Student Count for Wayne Country Regional Educational Service Agency, Detroit Public Schools Community District, Cass Technical High School, the School at Marygrove (Race/Ethnicity, Economically Disadvantaged, 2023–2024), accessed September 6, 2025, https://www.mischooldata.org/report‐builder/.

[19] Navigating the Health Care System , “Moving Health Care Upstream,” accessed December 31, 2025, https://www.movinghealthcareupstream.org/navigating‐the‐health‐care‐system/.

[20] A. Palmer and E. Singleton , “Creating the Healthiest Generations of Children: How Nemours Children's Health Is Increasing Accessibility to Health Education,” Delaware Journal of Public Health 10, no. 4 (2024): 70, 10.32481/djph.2024.10.16. PMC11526696 39493244

[21] L. Paakkari and O. Okan , “COVID‐19: Health Literacy Is an Underestimated Problem,” Lancet Public Health 5, no. 5 (2020): e249–e250, 10.1016/S2468-2667(20)30086-4. 32302535 PMC7156243

[22] E. Widnall , S. Dodd , A. E. Russell , et al., “Mechanisms of School‐Based Peer Education Interventions to Improve Young People's Health Literacy or Health Behaviours: A Realist‐Informed Systematic Review,” PLoS One 19, no. 5 (2024): e0302431, 10.1371/journal.pone.0302431. 38820530 PMC11142678

[23] M. Magerčiaková , A. Lochmannová , D. Mainz , and G. Debska , “Nursing Students' Perceptions of Health Literacy: A Cross‐Country Comparative Study,” Bratislava Medical Journal 126, no. 3 (2025): 351–357, 10.1007/s44411-025-00057-0.

[24] D. A. DeWalt and A. Hink , “Health Literacy and Child Health Outcomes: A Systematic Review of the Literature,” Pediatrics 124 (2009): S265–S274, 10.1542/peds.2009-1162B. 19861480

