# Supplementary material for: Exploratory Evaluation of a Medical Student‐Led Health Literacy Program Among High School Students
Source: J Sch Health. 2026 Aug 20;96(9):e70220. doi: 10.1111/josh.70220 (PMC13492087; doi:10.1111/josh.70220)
Supplement: Supplementary file 1 [file JOSH-96-0-s001.docx]

Supplemental Table 1. Sample Pre- and Post-Intervention Survey

| **Statement** | **Strongly Disagree** | **Disagree** | **Undecided** | **Agree** | **Strongly Agree** |
| --- | --- | --- | --- | --- | --- |
| 1. I know what services a Primary Care Provider can help me with. |  |  |  |  |  |
| 2. I am confident that I can identify what makes up a healthy diet. |  |  |  |  |  |
| 3. If someone is overdosing on an opioid, I am confident that I know how to help them. |  |  |  |  |  |
| 4. I am confident in recognizing the symptoms of an opioid overdose. |  |  |  |  |  |
| 5. I can explain what a PPO is. |  |  |  |  |  |
| 6. I can effectively explain what a deductible is. |  |  |  |  |  |
| 7. I know what adeno- means medically. |  |  |  |  |  |
| 8. I know what -megaly means medically. |  |  |  |  |  |
| 9. I can confidently find reputable sources of medical and health information. |  |  |  |  |  |
| 10. I can advocate for myself and my medical needs when meeting with my care team. |  |  |  |  |  |
